# Case of Occlusion of the Os-Uteri at Term, Resulting in Delivery through the Rectum

**Published:** 1874-11

**Authors:** Goronwy Owen


ATLANTA

### Medical and Surgical Journal.

Vol. XII.] NOVEMBER—1874. [No. 8.

### Original Communications.

### CASE OF OCCLUSION OF THE OS UTERI AT TERM, RESULTING IN DELIVERY THROUGH THE RECTUM.

By GORONWY OWEN, M.D.

*Read before the Medical Association of the State of Alabama, April,* 1874.

The patient in whom the occlusion of the os uteri occurred, which I am about to describe, was a mulatto woman, delicate in appearance, though she says she has always enjoyed good health. She was married in 1863, at the age of twenty-three, and had one child, which died soon after its birth, since which time she has lost flesh, and had some pain at each menstrual period, yet not sufficient to interfere with her household duties.

She was induced to accompany a lady, in the capacity of a nurse, in October, 1869, to Washington City, where she remained until October, 1870, when she returned to her husband at Mobile, much improved in flesh and appearance. She menstruated in October without pain, and again in November, the function being in every respect normal. On the 25th day of December, 1870, the time at which the catamenial flow should again have appeared, she was seized with an intense pelvic pain, evidently uterine, for the relief of which I was called in. I found her in great agony—without fever or any other symptom of inflammation. Opiates were prescribed freely, and after two or three days the pain subsided without the establishment of the menstrual flow. No vaginal examination was made, as the opinion was entertained that conception had taken place and that the pains were only threatenings of an abortion, which I believe was successfully resisted by the prompt and free administration of opium. From this time onward my patient presented the usual signs of pregnacy. She was not sick, but gradually emaciated until the 20th of August, 1871. When labor commenced, a colored midwife was sent for, who remained in attendance two days, and the labor not advancing satisfactorily, I was requested to visit the patient with the view of effecting an instrumental delivery. Upon entering the room, the bearing down pains induced the belief that labor had advanced to the second stage. Upon a vaginal examination the head was found upon the perineum. The presentation and position accurately made out, and it was thought that delivery was only prevented by a tough bag of waters.

A further re-examination, however, undeceived me, and revealed the true nature of the difficulty, as it became only too evident that what I had at first supposed was a dense membrane was in reality the attenuated wall of the vaginal portion of the uterus, upon which, after a most careful search, no os was found. The facts of the case were now related to my partner, Dr. Gaines, who visited the patient with me, and, after a careful examination, fully concurred with my opinion.

The case being a rare one and of much interest, Dr. Webb, an old and experienced obstetrican, was invited to visit the patient with us, and he too failed to find anything like an os uteri. Being aware of the rule to operate when, after a thorough digital and instrumental examination had been made, and no os found, Dr. Gilmore (a surgeon) was called in consultation. He, with Sims' speculum, brought into view the entire vaginal portion of the uterus, and upon the anterior part a depression or dimple was found, with œdematus edges, or lips. This, we inferred, was the original location of the os, and here we decided to make an opening, which was easily done. No hemorrhage followed the incision, but immediately dilatation resulted to the extent of 1½ inches, through which we could feel the child's head, covered with the amnion, thus encouraging the hope that the completion of the delivery could now be safely left to nature's efforts; but no progress being made for some time, it was thought proper to stimulate uterine action by ergot, which was accordingly given without avail, as the patient went to sleep and slept gently until the next morning—then all uterine action had ceased. Still it was thought that the contraction would again recur, and of course no interference was attempted. To our great surprise, the absence of uterine action continued until the next month—the period, perhaps, of the most regular menstrual discharge. At this time pains set in as in natural labor, just as they did in the first instance, though not so strong as before, nor did they make any perceptible impression on the os, as it maintained its former dilatation. Every endeavor was now made to induce the patient to permit us to use an artificial dilatation, but to no purpose. She and her friends positively opposed any kind of assistance.

The contractions again diminished in strength, and finally ceased altogether as before.

Our patient now passed another month of comparative comfort, feeling all the while the motions of the fœtus; finally, however, she began to suffer pain as before, which, after two or three days, passed off, as was the case on the two previous occasions.

After the third month the patient did not feel the fœtus move, nor could the sounds of the fœtal heart be detected, and it soon became evident that the uterus was getting smaller, the patient felt lighter, and was able to attend to her daily duties, and naturally enough felt convinced that her doctors had made a great mistake. Yet, upon a vaginal examination, the presentation and position could still be easily made out, and the patient and her friends were informed that there was a child in the womb, and that it was dead, and that sooner or later she would be compelled to have it extracted, and that to delay the operation would only increase the danger, as the opening in the womb was gradually diminishing, and would certainly close up again. This appeal to her fears was futile, as she was conscious of daily improvement in her general health. This fact, with her want of confidence in our opinion, made her strong in the belief that no harm could result from a cause which she and all her friends felt did not exist.

As time passed, the conviction of the truth of her opinion was strengthened, for she was well enough to walk about the city without inconvenience, and her size—from the absorption of liquor amnii and shriveling of the fœtus—had diminished so much as not to attract attention. The outline of the child's body could still be made out through the abdomen, and the head could be felt as distinctly as at any previous examination through the vagina.

This condition continued until July, 1873, when, after a long walk of $3\frac{1}{4}$ miles in the country and back, she had a chill followed by fever, which lasted nine days, and, though very sick, she did not complain of any uneasiness in the uterus, nor did pressure upon the abdomen or through the vagina cause any. At first it was doubtful whether the fever was caused by the encased child or was malarial, as she lived in a miasmatic district. In a week from the cessation of the fever, she had another chill, followed by fever, which continued to recur at irregular intervals, followed now with a sense of heat in the pelvis, and with considerable rectal trouble. An examination was now made (both digital and instrumental), and it was found that the bones of the cranium were separated, and that the incision which Dr. Gilmore made had nearly closed—in fact, I was unable to pass Emmet's uterine probe more than $\frac{1}{2}$ inch into the cervix, which was now much thickened by the results of inflammation.

The condition of my patient was now deplorable, and I again endeavored to persuade her to submit to an operation as the only possible chance for relief to her suffering, telling her no cutting would be required, as I thought I could, by using sponge tents, effect sufficient dilatation. Drs. Fournier and Moses were now called in, and, after careful examinations, warned the patient that if she persistently refused all assistance, she would or must die; to which she answered that her friends were unwilling and that she would have to die. Her agony was now beyond description. She emaciated rapidly, and had an offensive muco-purulent discharge from the rectum, attended with a constant bearing down effort. A rectal examination proved that an angle of one of the cranial bones had perforated the walls of the uterus and rectum; but as my patient still obstinately refused to allow any interference, I became discouraged and prescribed opium to relieve her pain, and abandoned her case, expecting every day to hear of her death. After a lapse of three weeks, however, she sent for me, stating that her suffering was intolerable, and that I might do what I pleased. An examination satisfied me that the entire fœtus could be extracted through the rectum, as one of the cranial bones was in the rectum, immediately over the anus, acting as a valve, and effectually preventing defecation. On the 29th of October I called upon Dr. Gilmore, and informed him that our patient was willing for us to deliver her in any way that we thought proper, provided we would give her chloroform, which in her condition was regarded as extremely hazardous, as she was literally skin and bones, with hardly a pulse at the wrist. It was thought that she would die under the operation, but it was her only chance, and we determined to give it to her. As a precaution, brandy was given liberally, and the chloroform administered. The pulse, if anything, improved under their joint influence, and we found that the great relaxation, from long and extreme emaciation and from the chloroform, permitted us to dilate the anus to almost any extent, and, without the least difficulty, to extract the fœtal mass; the greater part of which—the cranial bones, and those of the upper extremities and the upper part of the body—was in the rectum. The pelvis, with the viscera, which were a putrifying mass, and bones of the lower extremities, were still in the uterus; all, however, were promptly extracted, and the uterus firmly contracted upon the hand as it was withdrawn, and of course no hemorrhage followed. After the completion of the extraction the patient did not seem any weaker than before it was undertaken, though her feet and hands were cold and clammy. Every effort to bring about reaction, by external heat and stimulants, was resorted to, and after a doubtful period of a week, our patient began slowly to convalesce and to relish food; and notwithstanding she had a purulent discharge from the rectum for some time afterward, her improvement was steady, and in a month she was able to walk about the room. By the 17th of February she had regained her strength and was in every respect well; indeed, she menstruated freely and without the least pain. In March she menstruated again without the least discomfort. Being of course much perplexed, I prevailed upon the patient to allow me to make an examination, with the view of ascertaining the condition of the parts after the recovery, which was, in every sense of the word, complete. I found, by a rectal examination, that the fistulous opening into the rectum had healed without any stricture or contraction.

Upon a vaginal examination, the cervix uteri was of normal length, perfectly movable and of a healthy pink color; and the os, which before the extraction, from inflammatory enlargement and induration, would not admit a probe, now readily permitted the passage of Simpson's uterine sound.

In conclusion, I may state that the fœtal specimen is preserved in the museum of the Alabama Medical College.

